# Background and distribution of lobar microbleeds in cognitive dysfunction

**DOI:** 10.1002/brb3.856

**Published:** 2017-10-16

**Authors:** Hirofumi Matsuyama, Yuichiro Ii, Masayuki Maeda, Maki Umino, Yukito Ueda, Ken‐ichi Tabei, Hirotaka Kida, Masayuki Satoh, Akihiro Shindo, Akira Taniguchi, Ryosuke Takahashi, Hidekazu Tomimoto

**Affiliations:** ^1^ Department of Neurology Mie University Graduate School of Medicine Tsu Mie Japan; ^2^ Department of Advanced Diagnostic Imaging Mie University Graduate School of Medicine Tsu Mie Japan; ^3^ Department of Radiology Mie University Graduate School of Medicine Tsu Mie Japan; ^4^ Department of Rehabilitation Mie University Hospital Tsu Mie Japan; ^5^ Department of Dementia Prevention and Therapeutics Mie University Graduate School of Medicine Tsu Mie Japan; ^6^ Department of Neurology Kyoto University Graduate School of Medicine Kyoto Japan

**Keywords:** cerebral amyloid angiopathy, cerebral microbleeds, hypertensive vasculopathy, magnetic resonance imaging, memory clinic

## Abstract

**Objectives:**

Cerebral microbleeds (CMBs) are often observed in memory clinic patients. It has been generally accepted that deep CMBs (D‐CMBs) result from hypertensive vasculopathy (HV), whereas strictly lobar CMBs (SL‐CMBs) result from cerebral amyloid angiopathy (CAA) which frequently coexists with Alzheimer's disease (AD). Mixed CMBs (M‐CMBs) have been partially attributed to HV and also partially attributed to CAA. The aim of this study was to elucidate the differences between SL‐CMBs and M‐CMBs in terms of clinical features and regional distribution.

**Materials:**

We examined 176 sequential patients in our memory clinic for clinical features and CMB location using susceptibility‐weighted images obtained on a 3T‐MRI. The number of lobar CMBs in SL‐CMBs and M‐CMBs was counted in each cerebral lobe and their regional density was adjusted according to the volume of each lobe.

**Results:**

Of the total 176 patients, 111 patients (63.1%) had CMBs. Within the patients who had CMBs, M‐CMBs were found in 54 patients (48.6%), followed by SL‐CMBs in 35 (31.5%) and D‐CMBs in 19 (17.1%). The SL‐CMB group showed a significantly higher prevalence of family history of dementia, whereas the M‐CMB group showed an increasing trend toward hypertension and smoking. The prevalence of AD was significantly higher in the SL‐CMBs group, whereas the prevalence of AD with cerebrovascular disease was higher in the M‐CMBs group. The regional density of lobar CMBs was significantly higher in the occipital lobe in the M‐CMB group, whereas the SL‐CMB group showed higher regional density between regions an increasing tendency in the parietal and occipital lobe.

**Conclusion:**

The between‐group differences in clinical features and regional distribution indicate there to be an etiological relationship of SL‐CMBs to AD and CAA, and M‐CMBs to both HV and CAA.

## 1 INTRODUCTION

Cerebral microbleeds (CMBs) are defined as small hypointense foci <10 mm in diameter on magnetic resonance imaging (MRI) using T2*‐weighted gradient‐recalled echo or susceptibility‐weighted imaging (SWI). It has been increasingly acknowledged that the location of CMBs either in the lobar or nonlobar territories may reflect their underlying etiology. Strictly lobar CMBs (SL‐CMBs) are thought to be caused by cerebral amyloid angiopathy (CAA) frequently in patients with Alzheimer's disease (AD), whereas nonlobar CMBs (deep or infratentorial) are thought to be due to hypertensive vasculopathy (HV) (Greenberg et al., 2009; van Rooden et al., 2009). Mixed (deep/infratentorial with lobar) CMBs (M‐CMBs) are also thought to reflect HV (Greenberg et al., 2009; Vernooij et al., 2008). HV and CAA may synergistically contribute to the development of lobar CMBs (Cordonnier & van der Flier, 2011; Fazekas et al., 1999; Kim et al., 2016; Lee, Kim, Kim, Yoon, & Roh, 2007; Park et al., 2013; Smith et al., 2010).

However, little is known about the differences between SL‐CMBs and M‐CMBs in terms of clinical features and distributional patterns of lobar CMBs. In this study, we compared clinical features between patients with SL‐CMBs and M‐CMBs, and examined both distribution and density in our memory clinic.

## 2 MATERIAL AND METHODS

### 2.1 Subjects

We performed a retrospective analysis of our prospectively collected database of 213 patients in the memory clinic of our hospital from October 2011 to October 2013. Exclusion criteria were as follows: normal cognitive function, treatable dementia, insufficient neuropsychological assessments, and inadequate MRI examination. Thirty‐seven patients were excluded, resulting in a total of 176 patients (75 male, mean age: 75.1 ± 7.3 years) that were finally selected. This sample included 99 patients diagnosed with AD, 16 with AD with cerebrovascular disease (CVD), 29 with mild cognitive impairment (MCI), 12 with vascular dementia (VaD), 5 with dementia with Lewy bodies (DLB), 6 with frontotemporal dementia, and 9 with other disorders. All diagnoses were based on each preestablished criteria. For AD, we used the criteria for probable AD of the National Institute of Neurologic Disorders and Stroke‐Alzheimer Disease and Related Disorders Association (NINCDS‐ADRDA) (McKhann et al., 1984) and for AD with CVD by Bruandet et al. (2009), and for vascular dementia (VaD), the criteria for probable VaD of the National Institute of Neurological Disorders and Stroke‐Association Internationale pour la Recherche et l'Enseignement en Neurosciences (NINDS‐AIREN) (Román et al., 1993). For MCI, we used the general criteria of the International Working Group on MCI (Winblad et al., 2004), for DLB, the clinical criteria of the consortium on DLB (McKeith et al., 2005), for frontotemporal lobar degeneration, the Lund‐Manchester criteria for frontotemporal dementia, progressive nonfluent aphasia, and semantic dementia (Neary et al., 1998). The examination of clinical findings and neuropsychological tests were made by a team that specializes in dementia, including a neurologist and speech‐language‐hearing therapist. The patients’ average mini‐mental state examination (MMSE) score was 22.4 ± 4.3 (mean ± SD).

The demographic and clinical data were obtained through review of the medical records. The presence of hypertension, hyperlipidemia, and diabetes was determined based on prior medical diagnosis and treatment in all patients. Smoking was defined as a history of tobacco use. Family history of CVD and dementia were collected within the second degree based on medical records. We also determined whether oral antithrombotic drugs (antiplatelet and/or anticoagulant drugs) had been administered. The study was approved by the Ethical Review Board of Mie University Hospital.

### 2.2 MRI protocol and rating of CMBs

MRI was performed on a 3 Tesla MR machine (Achieva, Philips Medical System, Best, Netherlands) using an 8‐ or 32‐channel phased‐array head coil as described previously (Ii et al., 2013). The MRI protocol included T1‐weighted, T2‐weighted, and 3D‐FLAIR imaging, diffusion‐weighted imaging (DWI), and SWI. SWI was performed to detect CMBs because this sequence has been shown to be more reliable for CMB detection (Cheng et al., 2013). The details of the SWI were as follows: field of view, 230 mm; matrix, 320 × 251 (512 × 512 after reconstruction; in‐plane resolution, 0.45 mm × 0.45 mm); section thickness, 0.8 mm with overcontiguous slice; minIP with 5 mm, repetition time (ms)/echo time (ms), 22/11.5 (in‐phase), 36 (shifted); number of signals acquired, one; flip angle 20° and acquisition time, 4 min 53 s.

CMBs were defined as small hypointense foci <10 mm in size on SWI according to the STandards for ReportIng Vascular changes on nEuroimaging (STRIVE) consensus (Wardlaw et al., 2013). T2‐weighted images were analyzed simultaneously with SWI to rule out vessels and flow voids, which might mimic CMBs. CMBs were counted throughout the brain and their topographical distribution was classified as “deep,” “infratentorial,” or “lobar” according to the microbleed anatomical rating scale (MARS) (Gregoire et al., 2009). For this study, CMBs were categorized as follows: 1) SL‐CMBs, whereby CMBs were restricted to “lobar” locations, 2) M‐CMBs, whereby CMBs were in both “lobar” and “deep” and/or “infratentorial” locations. 3) Deep CMBs (D‐CMBs), whereby CMBs were only found in “deep” locations, 4) Infratentorial CMBs (I‐CMBs), whereby CMBs were only in “infratentorial” locations. Using the same way as MARS, we defined CMB distribution as follows; 1) “lobar” was the entire cerebral lobe including cortical/subcortical CMBs, 2) “deep” was the basal ganglia, thalamus, internal capsule, external capsule, corpus callosum, and deep and periventricular white matter, and 3) “infratentorial” was the brainstem and cerebellum.

To assess clustering effects of lobar CMBs in each cerebral lobe, we calculated the ratio of CMBs actual value (Observed) and expected lobar volume (Expected) using the methodology reported by Mesker et al. (2011).

### 2.3 Statistical analysis

Statistical analyses were performed using Windows SPSS software package version 23 (Chicago, Illinois). We used the χ^2^ test for categorical variables between‐group comparisons and the Shapiro–Wilk test and Mann–Whitney test for continuous variables. We used the binomial test to test whether the CMBs distributed in each lobe existed in proportion to the mean volume of that lobe. A significance level of *p *<* *.05 was applied in these comparisons.

## 3 RESULTS

The clinical characteristics of the 176 patients are shown in Table 1. Of the 176 patients, 111 (63.1%) had CMBs. There were no significant differences in sex, MMSE score, vascular risk factors excluding hypertension, family history of CVD or dementia, and the prevalence of antithrombotic therapy between CMBs positive and CMBs negative. However, age and prevalence of hypertension were significantly higher in patients with CMBs (*p *=* *.014, *p *=* *.040, respectively).

**Table 1:** Clinical backgrounds for CMBs(+) group versus CMBs(−) group

CMBs	CMBs (+)	CMBs (−)	*p*‐value
No. of patients^a^	*N *= 111	*N *= 65	
Age (years)	76.04 ± 6.79	73.41 ± 7.41	.014*
Male sex	50 (45.0%)	25 (38.5%)	.394
MMSE score	22.08 ± 4.61	23.03 ± 4.31	.190
HTN	57 (51.4%)	23 (35.4%)	.040*
DM	21 (18.9%)	14 (21.5%)	.674
HL	28 (25.2%)	20 (30.8%)	.425
Smoking	24 (21.6%)	12 (18.5%)	.616
Antithrombotic therapy	26 (23.4%)	13 (20.0%)	.598
CVD family history	14 (12.6%)	6 (9.2%)	.495
Dementia family history	17 (15.3%)	14 (21.5%)	.296
AD	56 (50.5%)	43 (66.2%)	.043*
(with CVD)	14 (12.6%)	2 (3.1%)	.034*
VaD	9 (8.1%)	3 (4.6%)	.375
MCI	20 (18.0%)	9 (13.8%)	.472
Others	12 (10.8%)	8 (12.3%)	.763

CMBs, cerebral microbleeds; MMSE, Mini‐Mental State Examination; HTN, hypertension; DM, diabetes mellitus; HL, hyperlipidemia; CVD, cerebrovascular disease; AD, Alzheimer's disease; VaD, vascular dementia; MCI, mild cognitive impairment.

a Number of patients is shown if not specified.

*p <* *.05 for CMBs(+) versus CMBs(−).

**Figure 1:**
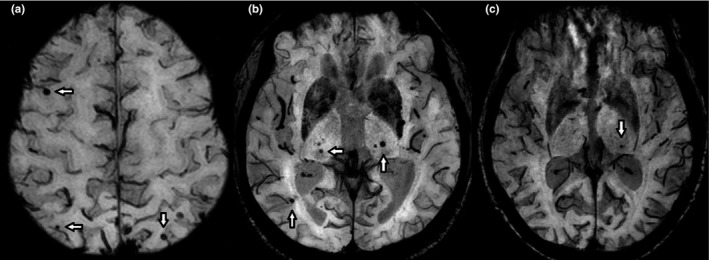
The representative examples of SL‐CMBs, M‐CMBs, and D‐CMBs. (a) AD 79‐year‐old‐male, SL‐CMBs, (b) VaD 79‐year‐old male, M‐CMBs, (c) VaD 82‐year‐old female, D‐CMBs

**Figure 2:**
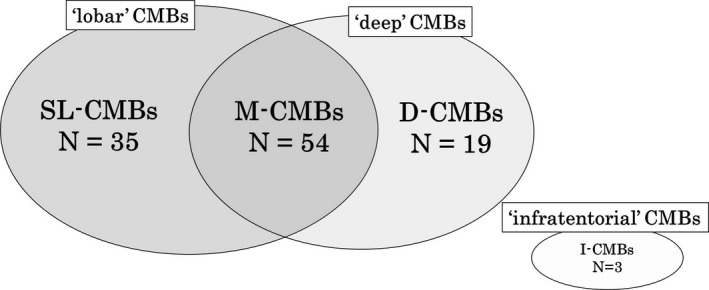
Distribution of CMBs for 176 cases, 111 cases (63.1%) are CMBs positive. CMBs = cerebral microbleeds

Figure 1 shows representative examples of SL‐CMBs, M‐CMBs, and D‐CMBs. Based on these classification, Figure 2 illustrates the distribution of CMBs. In terms of type of CMBs, M‐CMBs were most prevalent (*n *= 54, 48.6%) followed by SL‐CMBs (*n *= 35, 31.5%), D‐CMBs (*n *= 19, 17.1%), and I‐CMBs (*n *= 3, 2.7%). There were no patients with D/I‐CMBs. Compared with the SL‐CMBs group, the deep CMBs‐positive group (i.e., M‐CMBs and D‐CMBs) was significantly associated with hypertension (*p *=* *.034). According to the chi‐square test result, on three‐group comparison in the SL‐CMBs, M‐CMBs, and D‐CMBs groups (Table 2), the prevalence of hypertension, smoking, and family history of dementia tended to be different (*p *=* *.034, *p *=* *.026, *p *=* *.045; respectively). The SL‐CMBs group also showed a significantly higher prevalence of family history of dementia than the deep CMBs‐positive group (*p *=* *.015).

**Table 2:** Three‐group comparison: SL‐CMBs·M‐CMBs·D‐CMBs

CMBs type	SL‐CMBs	M‐CMBs	D‐CMBs	*p*‐value
No. of patients^a^	35	54	19	
Age (years)	74.86 ± 7.86	76.15 ± 6.02	77.68 ± 6.34	.404
Male sex	15 (42.9%)	26 (48.1%)	7 (36.8%)	.677
MMSE score	22.29 ± 4.80	22.09 ± 4.00	21.53 ± 5.97	.891
HTN	13 (37.1%)	29 (53.7%)	14 (73.7%)	.034*
DM	7 (20.0%)	8 (14.8%)	5 (26.3%)	.520
HL	10 (28.6%)	10 (18.5%)	6 (31.6%)	.390
Smoking	5 (14.3%)	17 (31.5%)	1 (5.3%)	.026*
Antithrombotic therapy	7 (20.0%)	16 (29.6%)	3 (15.8%)	.378
CVD family history	5 (14.3%)	7 (13.0%)	2 (10.5%)	.926
Dementia family history	9 (25.7%)	8 (14.8%)	0 (0.0%)	.045*
AD	24 (68.6%)	21 (38.9%)	9 (47.4%)	.023*
(with CVD)	1 (2.9%)	11 (20.4%)	2 (10.5%)	.052
VaD	2 (5.7%)	6 (11.1%)	1 (5.3%)	.579
MCI	4 (11.4%)	10 (18.5%)	5 (26.3%)	.378
Others	4 (11.4%)	6 (11.1%)	2 (10.5%)	.995

CMBs, cerebral microbleeds; MMSE, Mini‐Mental State Examination; HTN, hypertension; DM, diabetes mellitus; HL, hyperlipidemia; CVD, cerebrovascular disease; AD, Alzheimer's disease; VaD, vascular dementia; MCI, mild cognitive impairment.

a Number of patients is shown if not specified.

*p < .05 for SL‐CMBs versus M‐CMBs versus D‐CMBs.

Among the patients with lobar CMBs, there were no significant differences in the clinical features between SL‐CMBs and M‐CMBs; however, the prevalence of AD was significantly higher in the SL‐CMBs group, whereas the prevalence of AD with CVD was significantly higher in M‐CMBs group (Table 3).

**Table 3:** Comparison of “lobar” CMB‐positive cases:SL‐CMBs versus M‐CMBs

CMBs type	SL‐CMBs	M‐CMBs	*p*‐value
No. of patients^a^	35	54	
Age (years)	74.86 ± 7.86	76.15 ± 6.02	.579
Male sex	15 (42.9%)	26 (48.1%)	.625
MMSE score	22.29 ± 4.80	22.09 ± 4.00	.637
HTN	13 (37.1%)	29 (53.7%)	.126
DM	7 (20.0%)	8 (14.8%)	.523
HL	10 (28.6%)	10 (18.5%)	.267
Smoking	5 (14.3%)	17 (31.5%)	.066
Antithrombotic therapy	7 (20.0%)	16 (29.6%)	.311
CVD family history	5 (14.3%)	7 (13.0%)	.858
Dementia family history	9 (25.7%)	8 (14.8%)	.201
AD	24 (68.6%)	21 (38.9%)	.006*
(with CVD)	1 (2.9%)	11 (20.4%)	.018*
VaD	2 (5.7%)	6 (11.1%)	.385
MCI	4 (11.4%)	10 (18.5%)	.370
Others	4 (11.4%)	6 (11.1%)	.963

CMBs, cerebral microbleeds; MMSE, Mini‐Mental State Examination; HTN, hypertension; DM, diabetes mellitus; HL, hyperlipidemia; CVD, cerebrovascular disease; AD, Alzheimer's disease; VaD, vascular dementia; MCI, mild cognitive impairment.

a Number of patients is shown if not specified.

*p <* *.05 for SL‐CMBs versus M‐CMBs.

The total number of lobar CMBs in each cerebral lobe in the SL‐CMBs and M‐CMBs groups was greater in the frontal lobe, followed by the parietal lobe, temporal lobe, and occipital lobe. The M‐CMBs group had significantly more numerous lobar CMBs in each cerebral lobe than SL‐CMBs group using the binomial test. Most lobar CMBs were located in the frontal lobe in both the SL‐CMBs (38.1%) and M‐CMBs (32.5%) group. Compared with the expected distribution based on the volume of the lobes, lobar CMBs occurred more often in the occipital and parietal lobes in the SL‐CMBs group. On the other hand, lobar CMBs occurred significantly more often in the occipital lobe (*p *<* *.05), and significantly less often in the frontal lobe (*p *<* *.05) in the M‐CMBs group between region (Table 4).

**Table 4:** Distribution of CMBs on each lobe (SL‐CMBs versus M‐CMBs)

	No. of CMBs (Observed)			*p*‐value	Lobe vol. (Expected)	Density of CMBs	
	Total	SL‐CMBs	M‐CMBs			SL‐CMBs	M‐CMBs
Frontal	461	32 (38.1%)	429 (32.5%)	.002	40.6%	0.94	0.80*
Parietal	323	23 (27.4%)	300 (22.7%)	.001	22.6%	1.20	1.00
Temporal	316	13 (15.5%)	303 (23.0%)	<.001	22.8%	0.69	1.02
Occipital	304	16 (19.0%)	288 (21.8%)	<.001	13.9%	1.37	1.57*
Total	1404	84	1320				

*p <* *.05 for observed versus expected number of CMBs as the density.

## 4 DISCUSSION

In order to investigate the differences between SL‐CMBs and M‐CMBs in terms of clinical features and regional distribution of lobar CMBs, we examined 176 patients in our memory clinic. Our major findings were as follows. First, on the analysis in patients with CMBs, a family history of dementia was associated with the SL‐CMBs group, and a higher prevalence of hypertension was found in patients with only D‐CMBs group than SL‐CMBs and M‐CMBs groups. Second, patients with M‐CMBs had more numerous lobar CMBs in all the cerebral lobes than patients with SL‐CMBs. Finally, patients with SL‐CMBs showed relatively high density of lobar CMBs in the occipital lobe, followed by the parietal lobe, whereas the M‐CMBs group showed a significantly higher density of lobar CMBs in the occipital lobe than other regions.

CMBs are frequently identified in patients followed in memory clinics (Cordonnier et al., 2006; Goos et al., 2010). In line with previous studies, we identified a greater age and hypertension as risk factors associated with CMBs. Then the prevalence of family history of dementia was significantly difference on three‐group comparison.

Genetic association with CMBs has been assessed in recent systematic reviews and meta‐analyses. These studies have suggested that *ApoE* ε*4* is one of the risk factors for prevalence of CMBs, especially those of a lobar distribution (Maxwell et al., 2011; Shilling et al., 2013). Family history of dementia is also associated with an increased AD risk, independent of carrying the *ApoE* ε*4* allele (Scarabino, Gambina, Broggio, Pelliccia, & Corbo, 2016). Although we did not assess *ApoE* genotype in this study, the association between SL‐CMBs and family history of dementia might indicate the underlying genetic factors.

Few previous studies have described regional distribution of lobar CMBs. A community‐based study using SWI on 3T‐MRI showed that the occipital lobe had the most numerous CMBs followed by the frontal lobe (Chung et al., 2016). In patients with AD, CMBs have been found to be most numerous in the occipital lobe, followed by the temporal lobe, in studies using T2* on 1.5T‐MRI (Pettersen et al., 2008) or SWI on 3T‐MRI (Uetani et al., 2013). On the other hand, in patients with subcortical VaD, lobar CMBs were found to be most numerous in the temporal lobe, followed by the frontal lobe, using T2* on 1.5T‐MRI (Seo et al., 2007). However, these studies simply counted the number of lobar CMBs without distinguishing between SL‐CMBs and M‐CMBs.

It is now generally accepted that SL‐CMBs are related to CAA, whereas the etiology of lobar CMBs in patients with M‐CMBs remains uncertain. One previous study showed that lobar CMBs were found most frequently in the temporo‐occipital lobes in patients with hypertension‐related intracerebral hemorrhage (ICH), while they were more frequently found in the parietal lobe in patients with CAA‐related ICH (Lee et al., 2007). Another study with patients with cortico‐subcortical hemorrhage showed that lobar CMBs were most numerous in the parietal lobe in patients with CAA pathology (Doden et al., 2016). A hospital‐based study with patients undergoing MRI screening for neurological symptoms revealed that hypertension had a significant association with CMBs in the posterior cerebral artery area (Jia, Mohammed, Qiu, Hong, & Shi, 2014).

Most previous studies have merely counted the number of CMBs without considering their density in each cerebral lobe. In the population‐based Rotterdam Scan study, Mesker and colleagues evaluated clustering effects of CMBs while taking into account the volumetric differences in each lobe; the authors found that lobar CMBs (in both SL‐CMBs and M‐CMBs groups) occurred significantly more often in the temporal lobe between region (Mesker et al., 2011). A hospital‐based study that adjusted by lobe volume showed that CAA‐related ICH and microhemorrhage occurred preferentially in the temporal and occipital lobes (Rosand et al., 2005). In our study, SL‐CMBs showed a relatively high density in the occipital and parietal lobes, whereas M‐CMBs showed significantly higher density in the occipital lobe than other regions.

Some previous studies have shown that the posterior cerebral artery territory may be affected by CMBs, because this region is particularly susceptible to hypertension and breakdown of the blood–brain barrier (Jia et al., 2014; McKinney, Sarikaya, Gustafson, & Truwit, 2012). Therefore, occipital CMBs in the M‐CMBs group could have been caused by HV only, or alternatively, by HV with adjunct CAA. Synergistic effects of HV and CAA on the development of lobar CMB has indeed been reported in patients with M‐CMBs in radiological (Cordonnier & van der Flier, 2011; Fazekas et al., 1999; Park et al., 2013) and neuropathological studies (Ellis et al., 1996; Olichney et al., 1995; Thal, Ghebremedhin, Orantges, & Wiestler, 2003). In addition, the presence of multiple lobar CMBs in itself may reflect CAA, even if these are not SL‐CMBs (Benedictus et al., 2013; Mesker et al., 2011).

Autopsy studies have revealed that around 90% of AD cases are associated with CAA pathology of varying severity (Jellinger, 2002), and therefore, lobar CMBs in patients with AD are thought to be related mainly to CAA (Goos et al., 2009; Pettersen et al., 2008). In accordance with this information, frequency of diagnosis with AD was higher in the SL‐CMBs group than the M‐CMBs group, whereas the prevalence of AD with CVD was higher in the M‐CMBs group than the SL‐CMBs group in this study. The M‐CMBs group tended to have higher frequency of hypertension and a significantly greater number of lobar CMBs than the SL‐CMBs group. Moreover, lobar CMBs in the M‐CMBs group clustered mostly in the occipital lobe. Taken together, these findings indicate there to be contribution of HV to the pathogenesis of lobar CMBs in the M‐CMBs, but the role of CAA remains uncertain. Further studies are needed to evaluate these findings clinically and histopathologically.

Frequency of smoking was lower in D‐CMBs than SL‐CMBs and M‐CMBs in our study. A previous study has found that smoking is especially related to lobar CMBs (Goos et al., 2010), and taken together, our results may reflect the association between smoking and lobar CMBs. In our study, there was no significant association of antithrombotic therapy with presence of CMBs. The population‐based Rotterdam Scan study found that antiplatelet agent was related to the presence of CMBs, but anticoagulation was not (Darweesh et al., 2013; Vernooij et al., 2009). A systematic review including ICH and ischemic stroke/TIA found that both antiplatelet and warfarin were associated with presence of CMBs (Lovelock et al., 2010). In contrast, a study with asymptomatic elderly subjects showed no significant association of antithrombotic therapy with CMBs (Kim, Kwon, & Kwon, 2012). A meta‐analysis on the relationship between antiplatelet therapy and CMBs found that antiplatelet therapy was significantly associated with presence of CMBs in patients with stroke but not in stroke‐free individuals, and that the association was significant in patients from Asian countries but not in patients from European countries (LiuS, 2015). Considering these results altogether, it is necessary to note that patient population, antithrombotic drug, and the observation period of each study have varied between studies, and that the association between antithrombotic therapy and the risk of CMBs remains controversial. Therefore, we may assume that a lack of correlation between CMBs and the antithrombotic therapy may be attributable to a small number of cases with stroke history and antithrombotic treatment.

The detection of CMBs on SWI has been reported to be more sensitive at 3T MRI than at 1.5T MRI (Nandigam et al., 2009). In this study, the prevalence and number of CMB was higher than earlier reports using 3T SWI in memory clinic setting (Goos et al., 2011; Shams et al., 2015; Uetani et al., 2013). The differences in age and disease prevalence between earlier studies and ours may have also influenced these results. Indeed, incidence of CMBs increases with age (Vernooij et al., 2008). In addition, the prevalence and number of CMB are relatively high in patients with AD, MCI, and VaD (Cordonnier et al., 2006; Shams et al., 2015).

Our study has some limitations. First, the sample size was relatively small and statistical power was therefore limited. Second, we did not investigate *Apo E* genotype, which influences the spatial distribution of CMBs (Loehrer et al., 2014). Finally, we did not examine useful biomarkers for vascular amyloid deposition, including amyloid imaging and Aβ 40 and Aβ 42 values in cerebrospinal fluid (Dierksen et al., 2010; Renald et al., 2012). However, we think that these data are an essential first step to understanding the clinical implications of differences between SL‐CMBs and M‐CMBs. A further large‐scale study including the above investigations is warranted.

## 5 CONCLUSIONS

In conclusion, patients with SL‐CMBs tend to have a family history of dementia and a preferential distribution of lobar CMBs in the occipital and parietal lobes, which is suggestive of CAA. Patients with M‐CMBs tend rather to have hypertension and an accumulation of lobar CMBs in the occipital lobe.

## CONFLICT OF INTEREST

Authors declare no conflict of interest.
